# No Endospore Formation Confirmed in Members of the Phylum *Proteobacteria*

**DOI:** 10.1128/AEM.02312-20

**Published:** 2021-02-12

**Authors:** Polina Beskrovnaya, Doaa Fakih, Isabelle Morneau, Ameena Hashimi, Dainelys Guadarrama Bello, Shipei Xing, Antonio Nanci, Tao Huan, Elitza I. Tocheva

**Affiliations:** a Department of Microbiology & Immunology, The University of British Columbia, Vancouver, British Columbia, Canada; b Department of Stomatology, Université de Montréal, Montréal, Quebec, Canada; c Department of Chemistry, The University of British Columbia, Vancouver, British Columbia, Canada; University of Toronto

**Keywords:** endospores, *Firmicutes*, cryo-electron tomography, correlative light electron microscopy, whole-cell lipidomic analysis, EDX of storage granules, EDX analysis, *Rhodobacter johrii*, *Serratia marcescens*, correlative light and electron microscopy, storage granules

## Abstract

Endospore formation is used by members of the phylum *Firmicutes* to withstand extreme environmental conditions. Several recent studies have proposed endospore formation in species outside *Firmicutes*, particularly in Rhodobacter johrii and Serratia marcescens, members of the phylum *Proteobacteria*. In this study, we aimed to investigate endospore formation in these two species by using advanced imaging and analytical approaches. Examination of the phase-bright structures observed in R. johrii and S. marcescens using cryo-electron tomography failed to identify endospores or stages of endospore formation. We determined that the phase-bright objects in *R. johrii* cells were triacylglycerol storage granules and those in S. marcescens were aggregates of cellular debris. In addition, *R. johrii* and S. marcescens containing phase-bright objects do not possess phenotypic and genetic features of endospores, including enhanced resistance to heat, presence of dipicolinic acid, or the presence of many of the genes associated with endospore formation. Our results support the hypothesis that endospore formation is restricted to the phylum *Firmicutes*.

**IMPORTANCE** Bacterial endospore formation is an important process that allows the formation of dormant life forms called spores. Organisms able to sporulate can survive harsh environmental conditions for hundreds of years. Here, we follow up on previous claims that two members of *Proteobacteria*, Serratia marcescens and Rhodobacter johrii, are able to form spores. We conclude that those claims were incorrect and show that the putative spores in *R. johrii* and S. marcescens are storage granules and cellular debris, respectively. This study concludes that endospore formation is still unique to the phylum *Firmicutes*.

## Abstract (precis)

Bacterial endospore formation is an important process that allows the formation of dormant life forms called spores. Organisms able to sporulate can survive harsh environmental conditions for hundreds of years.

## INTRODUCTION

Spores represent a dormant state of bacteria that can persist for many years (1–3). Bacterial sporulation encompasses diverse modes; however, it is typically triggered by starvation and ultimately results in the production of metabolically inactive cells displaying increased resilience to stressors. For example, low nitrogen or carbon availability in *Firmicutes* can stimulate formation of endospores resistant to UV radiation, extreme pH, high temperature, and pressure (4–6). Similarly, exospore formation in *Actinobacteria* and fruiting-body production in *Myxococcus* have also been linked to nutrient limitation and can serve for preservation of genetic material under unfavorable environmental conditions (7–9). Despite the apparent similarities between these different types of sporulation, the underlying transformations are morphologically distinct and encoded by nonhomologous pathways (10).

Endospore formation begins with asymmetric cell division, with the septum placed near one pole of the cell, and produces two cells with different fates (11–13). Upon septation, the smaller compartment becomes engulfed through a phagocytosis-like mechanism, yielding a prespore bound by two lipid membranes in the cytoplasm of the mother cell. Subsequent endospore maturation involves the synthesis of protective layers, such as the peptidoglycan-based cortex and proteinaceous coat. Metabolic inactivation is achieved by gradual dehydration of the core through replacement of water with dipicolinic acid (DPA) and calcium ions and compaction of DNA with DNA-binding proteins. Together, these modifications account for the resistance properties of endospores (14). Ultimately, the spore is released upon lysis of the mother cell (15). In contrast, other modes of sporulation, such as those observed in *Actinobacteria* and *Myxococcus*, produce spores through morphological differentiation and cell division without engulfment.

Several studies in the past decade have reported, but not proven, formation of endospores in *Proteobacteria* (16, 17). While endosporulation has recently been confirmed in some Gram-negative bacteria, all of the identified organisms still belong to the phylum *Firmicutes*, highlighting the question of evolutionary origins of the bacterial outer membrane (18). Additionally, sporulation involves tight cooperation of hundreds of genes distributed across the chromosome, hindering acquisition of this pathway through horizontal gene transfer (10, 19, 20). Therefore, if confirmed, the ability to form endospores across distantly related bacterial phyla suggests an ancient nature of the process and can provide clues to the characteristics of the last bacterial common ancestor (10). In this study, we investigated two articles attributing sporulation to members of *Proteobacteria* (16, 17). Briefly, Girija et al. (17) described endospore production in the purple, nonsulfur bacterium Rhodobacter johrii, strain JA192(T), a close relative of the model organism for bacterial photosynthesis Rhodobacter sphaeroides. The second study, by Ajithkumar et al. (16), reported endospore formation in Serratia marcescens subsp. *sakuensis* (strain no. 9; KRED^T^), a pathogenic bacterium that infects humans and causes bacteremia, urinary tract infection, and wound infections (21). Thus, confirmation and further characterization of endospore formation in these organisms can bring valuable insight into the physiology of these species and the role of endospore formation in diversification and speciation of modern phyla.

In this study, we employed cutting-edge structural biology techniques, such as cryo-electron tomography (cryo-ET), correlative light and electron microscopy (CLEM), and energy-dispersive X-ray spectroscopy (EDX), as well as biochemical and microbiological approaches, to characterize endospore formation in *R. johrii* and S. marcescens. Our results showed that *R. johrii* and S. marcescens were unable to form endospores as previously reported (16, 17). Further analyses indicated that the putative spores in *R. johrii* were lipid storage granules (SGs) rich in triacyclglycerols (TAGs) and that the phase-bright objects in S. marcescens were aggregates of cellular debris. Overall, our observations contradict the previously published studies by Girija et al. and Ajithkumar et al. and support the observation that these members of *Proteobacteria* are unable to form endospores.

## RESULTS AND DISCUSSION

### Prolonged incubation induces formation of phase-bright objects in *R. johrii* and S. marcescens.

**FIG 1:**
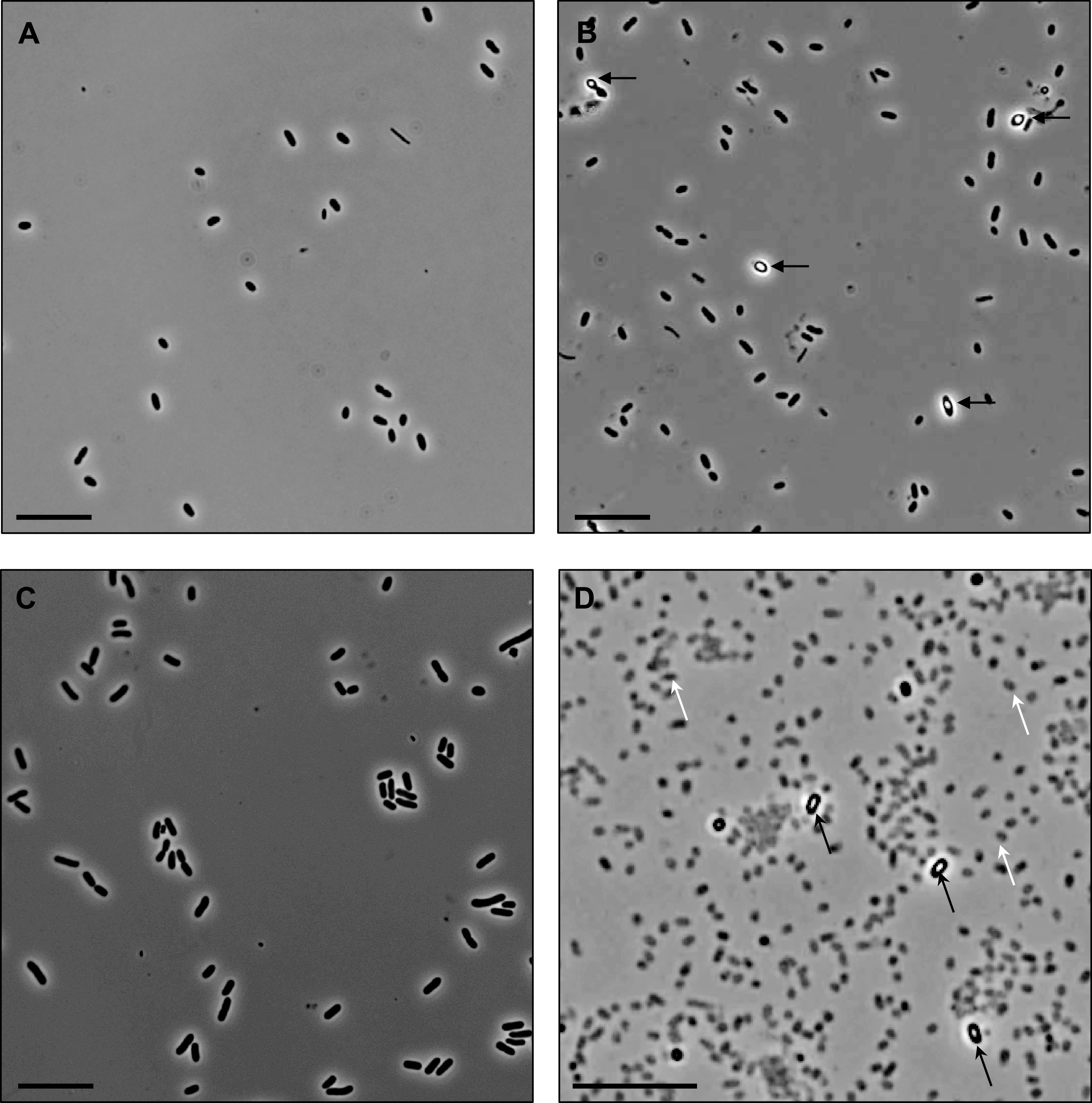
Phase-contrast light microscopy of *R. johrii* and S. marcescens cells. (A) Two-day-old *R. johrii* cells lack phase-bright objects. (B) Seven-day-old *R. johrii* cells show phase-bright objects (black arrows). (C) Seven-day-old S. marcescens cells lack phase-bright objects. (D) After 65 days, S. marcescens cells show two kinds of cell morphologies: phase-bright (black arrows) and ghost cells (white arrows). Scale bar, 10 μm.

For initial assessment of the previously reported endospore formation, we cultivated *R. johrii* and S. marcescens according to the published conditions and examined the cultures with phase-contrast light microscopy (LM) (Fig. 1). Vegetative *R. johrii* cells appeared phase-dark (Fig. 1A); however, after 7 s incubation, phase-bright objects were observed either at one pole or mid-cell (Fig. 1B, black arrows). Vegetative S. marcescens cells also appeared phase-dark (Fig. 1C). Although phase-bright objects were occasionally visible at mid-cell following a 65-day incubation (Fig. 1D, black arrows), the majority of culture was dead and appeared as “ghost” cells (Fig. 1D, white arrows). Altogether, our results recapitulate reports of formation of phase-bright objects in *R. johrii* and S. marcescens following extended incubation under nutrient-limited conditions.

### Characterization of phase-bright objects in *R. johrii*.

**FIG 2:**
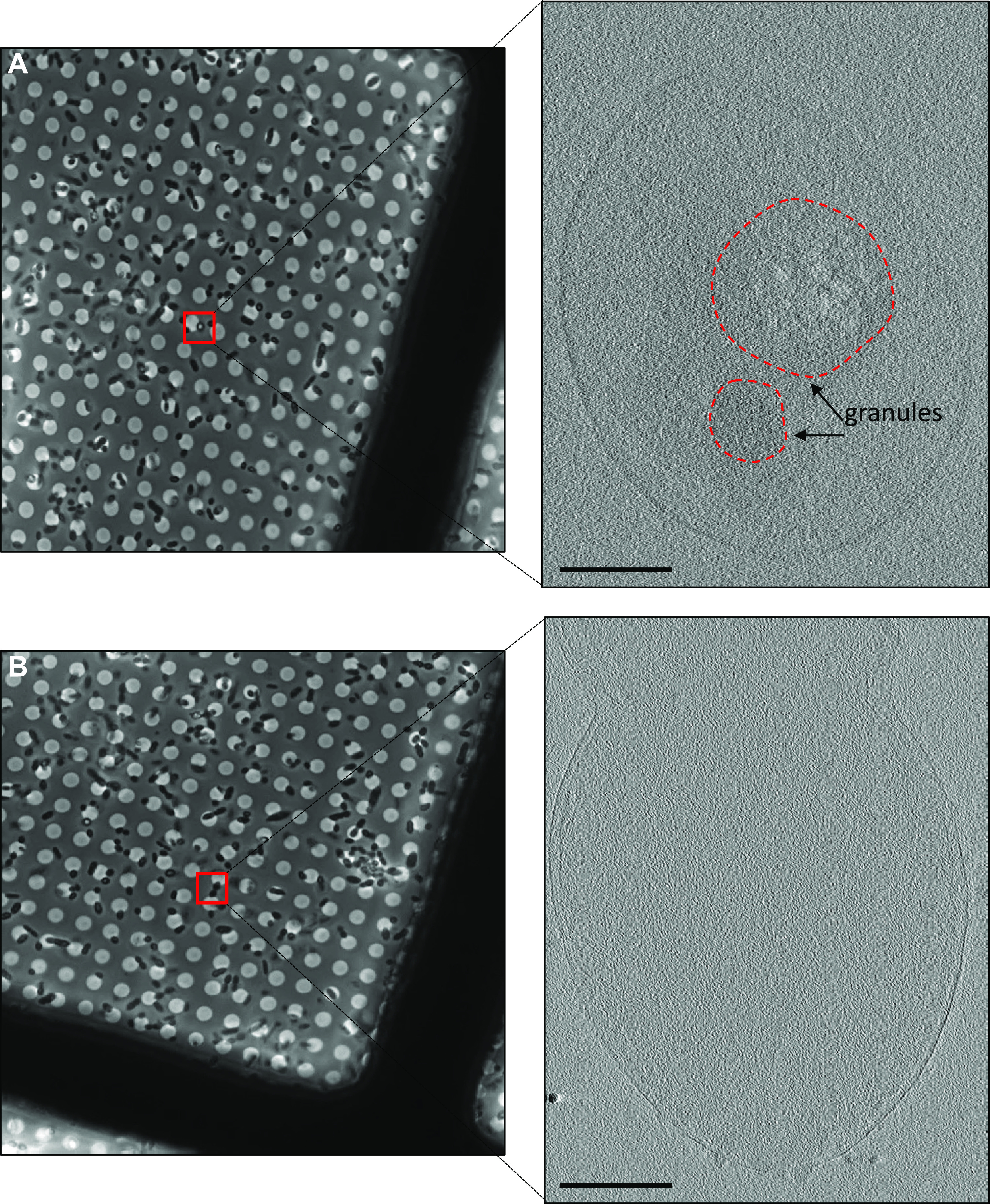
Correlative light and cryo-ET of *R. johrii.* (A) (Left) Phase-contrast microscopy image of an *R. johrii* cell (boxed) displaying a phase-bright object. (Right) Tomographic slice of the same cell showing two granular structures. (B) (Left) Phase-contrast microscopy image of *R. johrii* cells (boxed) lacking phase-bright objects. (Right) Tomographic slice of the same cell showing lack of subcellular structures. Scale bar, 200 nm.

To further characterize the phase-bright objects observed in *R. johrii*, we performed correlative LM and cryo-ET experiments on phase-bright and phase-dark cells following extended incubation (Fig. 2). Tomograms of *R. johrii* cells with phase-bright objects revealed the presence of intracellular granules, which were highly sensitive to the electron beam, as represented by the sample damage (Fig. 2A). Beam sensitivity was detected regardless of the total dose used (25 to 150 electrons [*e*^−^]/A^2^), suggesting that the granules were rich in lipids. Further, the spherical nature of the granules resembled previously characterized storage granules (SG) in bacterial cells (22). No evidence of sporulation-associated morphological changes, such as engulfing membranes or the presence of immature or mature spores in the sample (*n* = 40), was observed, indicating that the phase-bright objects were not endospores. Finally, cells with phase-bright objects always displayed 1 to 3 of the 100- to 250-nm-diameter granules (Fig. 2A), whereas the phase-dark cells lacked the presence of granules (Fig. 2B). Thus, our observations suggest that the phase-bright objects were likely lipid-containing SGs.

**FIG 3:**
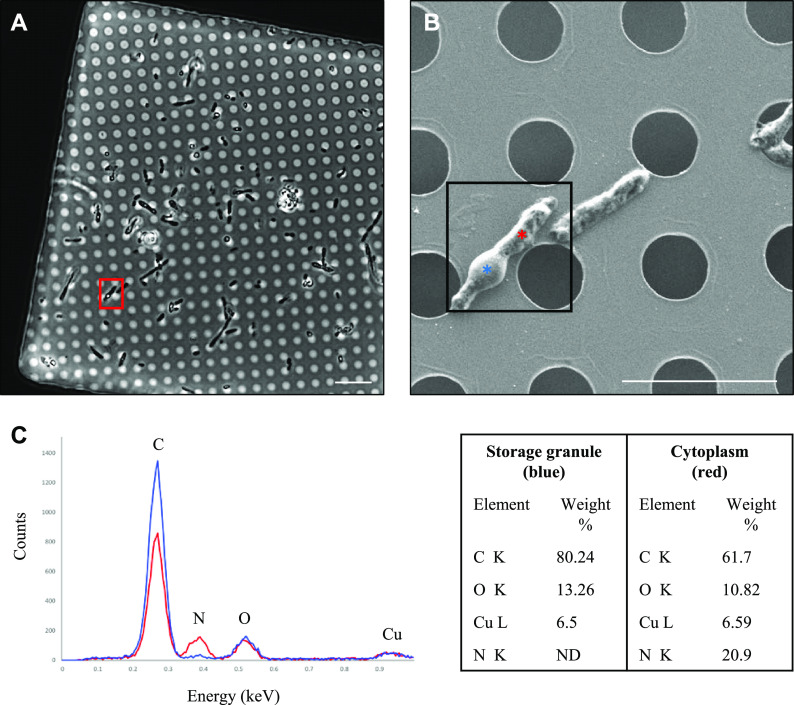
Correlative LM and SEM of *R. johrii* for storage granule characterization with EDX. (A) An LM image of *R. johrii* shows the presence of storage granules (phase-bright objects) inside a cell (red square). (B) The same cell as in panel A imaged with SEM. Areas corresponding to the storage granule and cytoplasm are depicted by blue and red asterisks, respectively. (C) Elemental composition of the storage granule (blue) and cytoplasm (red) using EDX semiquantitative analysis. Major peaks are assigned and data are summarized in a table format. Scale bars, 10 μm (A) and 5 μm (B). ND, nondetected.

To determine the composition of the storage granules, we performed correlative LM and scanning electron microscopy (SEM) in combination with EDX compositional analysis. Cells possessing the putative storage granules were identified with phase-contrast microscopy (Fig. 3A) and examined at higher resolution with SEM (Fig. 3B). Correlative LM and SEM were then used to guide EDX analysis, so that spectra were collected from a region containing the putative storage granules and a cytoplasmic region lacking the storage granules (Fig. 3C). Elemental analysis of the storage granule (blue spectrum) revealed counts for carbon (C) of 80.24%, oxygen (O) of 13.26%, and copper (Cu) of 6.5% (due to the copper of the EM grid). Cytoplasmic analysis (red spectrum) revealed lower counts for carbon (61.7%) and oxygen (10.82%), copper at 6.59%, and elevated counts for nitrogen (N; 20.9%) (Fig. 3C).

Based on the cryo-ET and EDX data, we hypothesized that the granules observed in *R. johrii* were composed of lipids, as lipids are enriched in carbon and oxygen atoms. To characterize the nature of the granular composition, we performed whole-cell lipidomic analysis of *R. johrii* 7-day-old cultures expressing phase-bright objects (granules) against *R. johrii* cultures grown for 2 days and lacking phase-bright objects as the negative control. Cultures producing putative storage granules where enriched in several lipids, the most abundant of which were triacylglycerols (TAGs) and phosphatidylethanolamines (PEs) (Table 1). Because PEs are typical membrane lipids, the increased levels observed under starvation conditions suggested that cells remodel their membrane composition to account for the environmental changes. TAGs are nonpolar, occur as insoluble inclusions in bacteria, and are considered a major source of energy (23, 24). TAGs have been shown to accumulate in actinobacteria and mycobacteria either as peripheral deposits associated with the cell envelope or as inclusion bodies in the cytoplasm (25). Previously, *in vitro* studies showed that mycobacteria accumulated TAG and wax ester when subjected to stresses, such as low oxygen, high CO_2_, low nutrients, and low pH (25–27). Similarly, we observed an increased propensity to form phase-bright objects in *R. johrii* cells incubated under low-nitrogen conditions in defined media. Therefore, it is likely that *R. johrii* utilizes TAG storage as an adaptive strategy in response to starvation, allowing cells to enter stationary phase and survive for longer periods. We thus conclude that the granules observed as phase-bright objects in *R. johrii* were storage granules enriched in TAGs.

**TABLE 1:** Lipidomic analysis of whole *R. johrii* cells

Lipid^*a*^	Lipid class	Fold change,^*b*^ R.j (+)/R.j (−)	*P* value
PE 33:1; PE 16:0-17:1^*c*^	PE	147.84	4.71E−09
TAG 58:1; TAG 16:0-24:0-18:1	TAG	93.81	1.81E−08
TAG 52:3; TAG 16:0-18:1-18:2	TAG	67.19	1.24E−08
TAG 54:5; TAG 18:1-18:2-18:2	TAG	60.41	1.37E−06
TAG 52:2; TAG 18:0-16:1-18:1	TAG	57.72	1.93E−07
TAG 54:4; TAG 18:1-18:1-18:2	TAG	54.46	2.37E−08
TAG 52:1; TAG 16:0-18:0-18:1	TAG	49.15	1.22E−10
TAG 56:2; TAG 16:0-18:1-22:1	TAG	48.89	5.37E−09
TAG 50:1; TAG 16:0-16:0-18:1	TAG	46.43	7.39E−09
TAG 54:2; TAG 18:0-18:1-18:1	TAG	44.82	2.37E−09
TAG 58:2; TAG 16:0-18:1-24:1	TAG	42.04	9.59E−09
TAG 52:2; TAG 16:0-18:1-18:1	TAG	41.06	5.93E−09
TAG 56:1; TAG 16:0-22:0-18:1	TAG	39.72	8.65E−10
TAG 54:1; TAG 18:0-18:0-18:1	TAG	39.35	1.15E−08
TAG 54:3; TAG 18:0-18:1-18:2	TAG	17.15	3.93E−08
TAG 50:2; TAG 16:0-16:1-18:1	TAG	12.30	1.90E−08
PE 32:0; PE 16:0-16:0	PE	11.55	1.73E−06
PC 39:3	PC	10.77	1.06E−08
PE 32:1; PE 16:0-16:1	PE	7.68	3.84E−07
TAG 48:1; TAG 14:0-16:0-18:1	TAG	4.94	2.00E−05
PE 35:2; PE 17:1-18:1	PE	3.09	6.92E−06
PC 36:4	PC	3.03	4.93E−06
TAG 48:1; TAG 16:0-16:0-16:1	TAG	2.52	4.51E−03
PC 32:1	PC	2.18	4.46E−07
PC 34:1; PC 16:0-18:1	PC	2.15	6.50E−08
DAG 36:2; DAG 18:1-18:1	DAG	2.08	5.34E−08
PC 34:2; PC 16:1-18:1	PC	2.02	1.57E−06

a Abbreviations: PE, phosphatidyl ethanolamine; TAG, triacylglycerol; PC, phosphatidylcholine; DAG, diglyceride.

b The total lipid composition of *R. johrii* expressing storage granules [R.j (+)] was compared to that of a fresh *R. johrii* culture lacking storage granules [R.j (−)].

c PE is a lipid class with two acyl chains. PE 16:0-17:1 indicates that the chain lengths are 16 carbons and 17 carbons and the saturation degrees are 0 and 1, respectively. PE 33:1 is the simpler form of PE 16:0-17:1.

### Characterization of phase-bright objects in S. marcescens.

**FIG 4:**
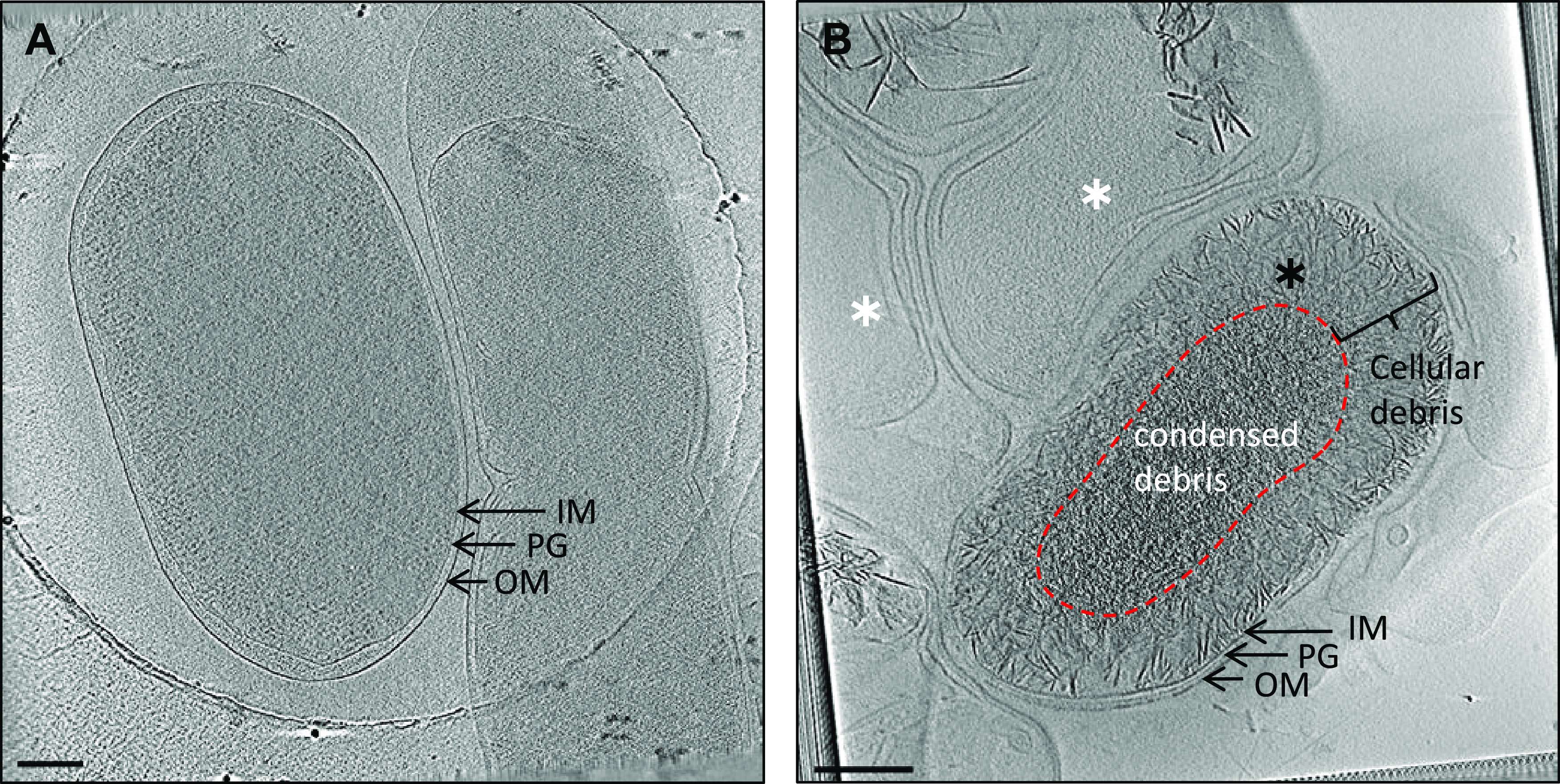
Cryo-ET of S. marcescens. Tomographic slices through the following are shown: vegetative cells from a 2-day-old culture (A) and cells from a 65-day-old culture showing phase-bright objects (B). Panel B shows two cell types: cells with accumulated cellular debris (black asterisk) and ghost cells void of cellular material (white asterisks). Scale bar, 200 nm. IM, inner membrane; PG, peptidoglycan; OM, outer membrane.

Tomograms of S. marcescens were collected for 2-day and 65-day-old cultures (Fig. 4). At 2 days, we observed regular morphology of vegetative cells, displaying cell envelope architecture typical for Gram-negative bacteria (Fig. 4A). S. marcescens grown for 65 days revealed the presence of two kinds of morphologies: cells packed with cellular debris (black asterisk in Fig. 4B) and cells void of any cellular material (white asterisks in Fig. 4B), likely correlating to cells containing phase-bright objects and ghost cells identified using LM, respectively. Extensive survey of the sample (*n* = 80) did not reveal any cells possessing intracellular membranes or morphologies suggestive of engulfing membranes or stages of sporulation. Neither of the two identified morphologies displayed any features similar to a cortex or proteinaceous spore coat characteristic of mature endospores. Additionally, we did not observe accumulation of storage granules within cells. Together, these results suggest that the appearance of phase-bright objects in S. marcescens was the result of accumulation of cellular debris and dehydration.

### *Proteobacteria* do not possess features of endospores following extended incubation.

Independent of imaging-based methods, endospores have traditionally been identified in samples through heat resistance and increased concentration of intracellular DPA. To verify the results of our cryo-ET experiments, we first investigated the heat resistance properties of *R. johrii* and S. marcescens following prolonged incubation and subsequent exposure to high temperatures. Despite an extended recovery period (7 days), no viable *R. johrii* cells were observed on solid media following 15, 30, and 60 min of incubation at 80°C. Similarly, viable cells were not isolated from S. marcescens cultures incubated at 60°C for 15, 30, and 60 min. In contrast, Bacillus subtilis cultures producing endospores and treated at 80°C for 15, 30, and 60 min yielded viable growth on solid media after a 24-h recovery period. Thus, we were unable to replicate the results of Girija et al. and Ajithkumar et al., who found viable cells following heat treatment of *R. johrii* at 80°C for 20 min and S. marcescens at 60°C for 15 min, respectively. Additionally, we quantitatively analyzed the presence of DPA in cultures of *R. johrii* and S. marcescens displaying phase-bright objects using a colorimetric method. Whereas the purified endospores of B. subtilis contained 6.74 μg/ml of DPA, no detectable amounts of DPA were observed in *R. johrii* or S. marcescens after prolonged cultivation. Collectively, these results indicate that *R. johrii* and S. marcescens cells do not possess the classic phenotypic features that are associated with endospore formation.

### Minimal subset of genes required for endospore formation not conserved in *R. johrii* and S. marcescens.

Endospore formation relies on expression of hundreds of conserved genes in a highly regulated manner (20, 28–30). For example, over 500 genes have been previously implicated in sporulation in the model firmicute B. subtilis (28). However, establishment of the minimal subset of genes required for endospore formation remains elusive, as many of the identified targets carry out redundant functions, e.g., histidine kinases, or are part of general pathways loosely associated with sporulation, such as iron uptake and DNA repair proteins (31). Consistently, several homologs to genes linked to sporulation have been detected in other phyla, including *Proteobacteria*, but have been shown to play regulatory roles in distinct processes, such as cell division and development (32, 33). Hence, possession of genes annotated as sporulative should not be considered concrete evidence to support sporulation capacity in a given species (5). Nevertheless, we investigated the genomes of *R. johrii* and S. marcescens for presence of genes that are conserved among all spore-forming bacilli and clostridia based on the COGs database and have been shown to play pivotal roles in endospore formation through functional studies (Table 2) (5, 34, 35). Our analysis showed that both *R. johrii* and S. marcescens completely lack the SpoIIDMP peptidoglycan remodeling complex required for spore cortex formation, the SpoIIQ-SpoIIIAA-AH channel complex involved in communication between the mother cell and the prespore and facilitating regulation of endospore maturation, and the major protein coat assembly components, such as SpoIVA (12, 14, 36). Further, the master regulator of sporulation encoded by all endospore formers, Spo0A, is absent in *R. johrii* and S. marcescens. Both strains also lack homologs to all five sporulation sigma factors, SigE, SigF, SigG, SigH, and SigK. Finally, *R. johrii* and S. marcescens do not possess DapB, required for production of dipicolinic acid in *Bacilli*, which plays a major role in dehydration of the spore core and, therefore, resistance and dormancy (14). A homolog of *etfA* (a gene associated with DPA synthesis in some *Clostridia*), however, was identified in *R. johrii* and Escherichia coli but not in S. marcescens (Table 2). The role of this gene in *Proteobacteria* has not been characterized, and it could encode an electron transfer flavoprotein. Overall, our analysis confirms the lack of the major structural and regulatory sporulation genes in the genomes of *R. johrii* and S. marcescens. In addition, Ajithkumar et al. were also unable to detect genes related to endospore formation in S. marcescens (16).

**TABLE 2:**
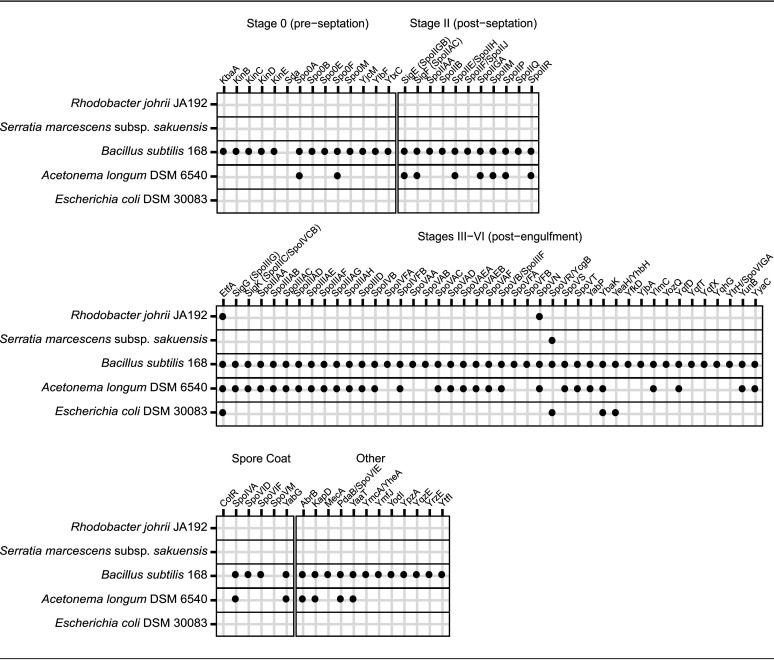
Analysis for presence of endospore formation genes in *R. johrii* and S. marcescens

### Concluding remarks.

Although endospore formation is considered a hallmark of the *Firmicutes* phylum (4, 5, 37), endospore production had been reported outside *Firmicutes*, particularly for two members of the phylum *Proteobacteria* (16, 17). These findings may affect our understanding of the evolutionary events surrounding outer membrane biogenesis and the significance of endospore formation in cell differentiation. In this study, using cutting-edge microscopy techniques and biochemical, microbiological, and bioinformatics approaches, we showed that the phase-bright objects observed in *R. johrii* and S. marcescens are storage granules and cellular debris, respectively. We did not observe mature spores or stages of endospore formation *in vivo*, and we failed to detect the pivotal biochemical and genomic features of endospore-producing bacteria in these organisms. Our findings thus demonstrate that *R. johrii* and S. marcescens are unable to form true endospores, which is in contrast to the results described by Girija et al. (17) and Ajithkumar et al. (16). Since we used the most advanced imaging techniques currently available to study whole-cell bacteria and their ultrastructure, previous results could be due to the presence of contamination with spore-forming bacteria or misinterpretation of methodology artifacts.

## MATERIALS AND METHODS

### Bacterial strains and growth conditions.

*R. johrii* and S. marcescens cells were purchased from the Leibniz-Institut DSMZ bacterial strain collection. *R. johrii* JA192 cells (DSMZ 18678) were cultivated as previously described by Girija et al. (17). Briefly, cells were grown aerobically at room temperature in R. sphaeroides solid and liquid media comprising 4 mM KH_2_PO_4,_ 1 mM MgCl_2_·6H_2_O, 7 mM NaCl, 22 mM NH_4_Cl, 0.04 mM CaCl_2_·2H_2_O, 17 mM sorbitol, 28 mM sodium pyruvate, 1.5 mM yeast extract, 1 liter distilled water (pH 7.0), 1 ml of trace element solution SL7, and 20 ng of vitamin B_12_ solution for 2 days for vegetative cells or 7 days to induce production of phase-bright objects. Additionally, cells were grown in Luria-Bertani (LB) broth at 30°C with agitation for 2 days and either harvested as the vegetative growth control or subsequently inoculated 1:100 into modified M9 medium for an additional 7 days to induce formation of phase-bright objects. The modified M9 medium contained 47.8 mM Na_2_HPO_4_, 22 mM KH_2_PO_4_, 8.56 mM NaCl, 18.7 mM (3.74 mM for limited nitrogen) NH_4_Cl, 1 mM MgSO_4_, 0.3 mM CaCl_2_, 0.4% (wt/vol) glucose, 1 μg/liter of biotin, 1 μg/liter of thiamine, 31 μM FeCl_3_·6H_2_O, 12.5 μM ZnCl_2_, 2.5 μM CuCl_2_·2H_2_O, 2.5 μM CoCl_2_·2H_2_O, 5 μM MnCl_2_·4H_2_O, and 2.5 μM Na_2_MoO_4_·2H_2_O. S. marcescens cells (DSMZ 30121) were cultivated in LB broth at 32°C with shaking at 200 rpm, as previously described by Ajithkumar et al. (16), for 7 days for vegetative growth or 65 days to induce formation of phase-bright objects. B. subtilis strain PY79 was chosen as the positive control for endospore formation, and cells were cultivated in LB broth at 37°C with shaking at 200 rpm overnight for vegetative growth or for 3 days to induce sporulation. LB agar was used for cultivation on plates for S. marcescens and B. subtilis.

### Detection of phase-bright objects using phase-contrast light microscopy.

*R. johrii* and S. marcescens cultures were pelleted and washed with 1× phosphate-buffered saline (PBS; pH 7.4) composed of 137 mM NaCl, 27 mM KCl, 10 mM Na_2_HPO_4_, and 1.8 mM KH_2_PO_4_. Cells were imaged with an upright Zeiss Axio Imager M2 microscope (Carl Zeiss, Oberkochen, Germany) equipped with a 506 monochrome camera and a 100× oil lens objective with a numerical aperture (NA) of 1.46.

### Sample preparation for correlative light and cryo-electron tomography.

*R. johrii* cells were lightly fixed using 2.5% paraformaldehyde in 30 mM phosphate buffer for 15 min, washed twice, and resuspended in 150 mM phosphate buffer. Bacterial cells were loaded onto Cu Finder R 2/2 EM grids (Electron Microscopy Sciences, Hatfield, PA), coated with 1 mg/ml of poly-l-lysine, and subsequently imaged at room temperature as described above. Following room temperature light microscopy, 20-nm colloidal gold particles (UMC Utrecht, Netherlands) were added and samples were plunge-frozen into liquid ethane-propane mix cooled at liquid nitrogen temperatures with a Mark IV Vitrobot (Thermo Fisher Scientific), maintained at room temperature and 70% humidity. Cryo-ET was conducted on cells with phase-bright signal as described below. For standalone cryo-ET experiments, samples were directly mixed with 20-nm colloidal gold particles, loaded onto glow-discharged carbon grids (R2/2; Quantifoil), and plunge-frozen as described above.

### Cryo-ET data collection.

For both standalone cryo-ET and CLEM experiments, tilt series were collected on a 300-kV Titan Krios transmission electron microscope (Thermo Fisher Scientific) equipped with a Falcon 2 camera. Tilt series were collected at nominal magnifications of ×14,000 to ×18,000, 1- to 3-degree oscillations, and a final dose of 30 to 150 *e*^−^/A^2^. Three-dimensional reconstructions were calculated with IMOD software package using the weighted back projection method (38).

### Correlative LM and SEM with EDX analysis.

*R. johrii* cells were fixed with 12.5% paraformaldehyde in 150 mM sodium phosphate buffer (73.6 mM K_2_HPO_4_, 26.4 mM KH_2_PO_4_; pH 7.5) and then washed three times with 150 mM sodium phosphate buffer (39). Glow-discharged Cu R2/2 grids were coated with poly-l-lysine hydrobromide solution (1 mg/ml) and dried for 30 min at 60°C. Fixed cells were loaded onto the grids and immediately imaged with LM in 1× PBS to identify phase-bright objects. The grids were subsequently air dried, and regions of interest identified with LM were examined with SEM using a JSM-7400F field emission scanning electron microscope (JEOL Ltd., Tokyo, Japan) operated at 5 kV without any coating. EDX analysis with a silicon drift detector (Octane, EDAX Inc., Mahwah, NJ) at 10 kV was used for semiquantitative elemental analysis of regions of interest.

### Whole-cell lipidomic analysis of *R. johrii*.

*R. johrii* cells displaying phase-bright properties were cultivated as described above, harvested by centrifugation (20 min at 5,000 rpm), and washed twice in sterile H_2_O. Cell pellets were then lyophilized overnight and stored at room temperature for up to 7 days. *R. johrii* cultures grown in LB for 2 days and lacking phase-bright objects were chosen as the negative control. Sample normalization between samples of *R. johrii* expressing storage granules [R.j (+)] and *R. johrii* lacking storage granules [R.j (−)] was achieved by their culture weights. For R.j (+) samples, 4.0 mg of cells (wet weight) was used; for R.j (−) samples, 2.5 mg was used. The lipidomic results were normalized according to culture weights. For the whole-cell lipidomic analysis, a methyl *tert*-butyl ether (MTBE)-based membrane lipid extraction protocol was used, with modifications (40). Briefly, samples in 1.5-ml Eppendorf vials were first mixed with 300 μl of ice-cold methanol and 10-μl internal standards. The mixture was then sonicated in ice-water bath for 15 min for protein precipitation. One milliliter of MTBE was added to the mixture, followed by vortex mixing for 20 min at room temperature for thorough lipid extraction. Next, 200 μl of liquid chromatography-mass spectrometry (LC-MS)-grade water was added to induce phase separation, and the samples were further mixed for 30 s. After settling for 10 min, the upper layer, containing the lipids, was transferred to new Eppendorf vials. To dry the lipid samples, the solvent was evaporated using a vacuum concentrator at 4°C. A total of 100 μl of isopropanol/acetonitrile (1:1, vol/vol) was added to reconstitute the dried residue. The reconstituted solution was vortexed for 30 s and centrifuged at 14,000 rpm at 4°C for 15 min. The resulting supernatants were transferred to glass inserts for LC-tandem MS (LC-MS/MS) analysis. Only lipids above the noise level (1,000 average intensity) were considered in the analysis. A cutoff value of at least 2× increase in average intensity and a *P* value threshold of 0.01 were used to determine significant increase in lipid species.

### Heat inactivation and counting of endospores.

After induction of the phase-bright objects in *R. johrii*, S. marcescens, and B. subtilis as described above, cells were washed with sterile, deionized water, spun at 10,000 × *g* for 10 min, and resuspended in chilled water. Suspensions of *R. johrii* and B. subtilis were heated to 80°C, and S. marcescens to 60°C, for 15 min, 30 min, and 1 h, as described previously (16, 17). After the heat treatment, the samples were centrifuged for 10 min at 10,000 × *g*. The pellets were washed five times to remove cellular debris and then plated onto solid media. *R. johrii* was incubated at 30°C for 7 days, S. marcescens was incubated at 32°C for 7 days, and B. subtilis was incubated at 37°C overnight, and plates were subsequently examined for viable growth.

### DPA detection.

Following the detection of phase-bright objects, dipicolinic acid (DPA) was detected as previously described (41). Briefly, 5-ml cultures of *R. johrii*, S. marcescens, and B. subtilis containing ∼10 mg of cells (dry weight) were autoclaved for 15 min at 15 lb/in^2^. The suspensions were cooled to ambient temperature, acidified with 0.1 ml of 1.0 N acetic acid, and incubated for 1 h to cluster the insoluble material. To remove cellular debris, the suspensions were centrifuged at 1,500 × *g* for 10 min. To each 4 ml of supernatant, 1 ml of 1% Fe(NH_4_)_2_(SO_4_)_2_·6H_2_O and 1% ascorbic acid in 0.5 M acetate buffer (pH 5.5) was added. Colorimetric shift at 440 nm was compared to a standard curve prepared with pure DPA (Sigma-Aldrich, Oakville, Canada).

### Detection of genes required for endospore production.

We assessed the genomes of S. marcescens subsp. *sakuensis* KRED^T^ and Rhodobacter johrii JA192—the two strains originally described by Girija et al. and Ajithkumar et al., respectively (16, 17)—for endospore formation genes. Using the recently updated COGs database, we compiled a list of genes associated with sporulation (5, 42). Corresponding KEGG and TIGRFAM hidden Markov models (HMMs) were used to assess the genomes for the presence of these genes using HMMER 3.2.1 (http://hmmer.org/) (43, 44). Genes without representative HMMs were identified using local alignment against sequences from B. subtilis 168 and Clostridium perfringens SM101 using a minimum percent identity of 30% and an E value of 1E−25. B. subtilis 168 (monoderm sporulator), Acetonema longum DSM 6540 (diderm sporulator), and Escherichia coli DSM 30083 (diderm nonsporulator) were included as sporulating and nonsporulating controls.
